# Mental Health Inequalities in Adolescents Growing Up in Post-Apartheid South Africa: Cross-Sectional Survey, SHaW Study

**DOI:** 10.1371/journal.pone.0154478

**Published:** 2016-05-03

**Authors:** Jayati Das-Munshi, Crick Lund, Catherine Mathews, Charlotte Clark, Catherine Rothon, Stephen Stansfeld

**Affiliations:** 1 Department of Health Services & Population Research, Institute of Psychiatry, Psychology & Neuroscience, King’s College London, London, United Kingdom; 2 Alan J Flisher Centre for Public Mental Health, Department of Psychiatry and Mental Health, University of Cape Town, Cape Town, South Africa; 3 Health Systems Research Unit, South African Medical Research Council, Cape Town, South Africa; and Adolescent Health Research Unit, Department of Psychiatry and Mental Health, University of Cape Town, Cape Town, South Africa; 4 Centre for Psychiatry, Wolfson Institute of Preventive Medicine, Barts and the London School of Medicine, Queen Mary University of London, London, United Kingdom; 5 St George’s, University of London, London, United Kingdom; University of Stellenbosch, SOUTH AFRICA

## Abstract

**Background:**

South Africa is one of the most ‘unequal’ societies in the world. Despite apartheid ending more than 20 years ago, material inequalities remain interwoven with ethnic/racial inequalities. There is limited research on the prevalence/predictors of common mental disorders (CMD) among young people. Adolescence is a unique time-point during which intervention may lead to improved mental health and reduced social problems later. The study objective was to assess mental health disparities in a representative sample of adolescents growing up in South Africa.

**Methods:**

Cross-sectional associations of race/ethnicity and material disadvantage with CMD and Post Traumatic Stress Disorder (PTSD) were assessed in a stratified random sample representative of school-attendees, aged 14–15 years, in a large metropolitan area of Cape Town. Validated instruments assessed mental disorders; these included: Harvard Trauma Questionnaire (PTSD); Short Moods and Feelings Questionnaire (depression); Zung self-rated anxiety scale (anxiety). Self-ascribed ethnicity was determined using procedures similar to the South African census and previous national surveys.

**Results:**

Response rate was 88% (1034 of 1169 individuals). Adolescents experienced a high prevalence of depression (41%), anxiety (16%) and PTSD (21%). A gradient between material disadvantage and CMD/ PTSD was evident across all ethnic/racial groups. Respondents self-identifying as ‘black’ or ‘coloured’ were disadvantaged across most indicators. After adjusting for confounders, relative to white children, relative risk (RR) of CMD in black children was 2.27 (95% CI:1.24, 4.15) and for PTSD was RR: 2.21 (95% CI:1.73, 2.83). Relative risk of CMD was elevated in children self-identifying as ‘coloured’ (RR: 1.73, 95% CI:1.11, 2.70). Putative mediators (violence, racially motivated bullying, social support, self-esteem) partially accounted for differences in CMD and fully for PTSD.

**Conclusions:**

Adolescent mental health inequalities in Cape Town are associated with material disadvantage and self-identification with historically disadvantaged groups.

## Introduction

Despite a growing body of evidence supporting linkages between poverty and common mental disorders in adults living in low and middle income countries (LMICs)[1], there remains a surprising dearth of evidence examining this for adolescents within these settings[2]. Globally, mental disorders contribute significantly to Disability-Adjusted Life Years (DALYs) and may be prevalent in up to a quarter of young people[2, 3]. In South Africa, the adjusted prevalence of mental disorders in adolescence has been estimated to be between 15–17%[2, 4]. This is lower than prevalence estimates from More Economically Developed Countries [2] although higher than prevalence rates reported from other LMIC settings[5].

The presence of mental disorders in adolescence is a strong predictor for mental health problems in adulthood[3, 6] and may have a direct impact on economic and educational outcomes, as well as crime and suicide[2]. Adolescents who live in households and attend schools which are socially and economically disadvantaged are at a greater risk of mental disorders through a number of mechanisms, including increased exposure to violence, food and income insecurity, substance abuse, reduced economic protection and fewer employment or career prospects[2]. In South Africa, some of these risk factors, such as exposure to violence and poverty are more prevalent[4, 5]. However, little work has been done to assess the association of these experiences with mental disorders, within this context[5].

From 1948 until 1994 the apartheid regime in South Africa led to a racially segregated society in which access to economic resources and medical care, as well as educational and employment opportunity were severely restricted for people socially classified as ‘non-white’. Although following the end of apartheid there were dramatic changes in the constitution including the right to health for all as a fundamental principle[7, 8], investigators conducting research in the post-apartheid era continued to report widespread disparities in health and access to healthcare across ethnic groups[7, 9–11] with health inequalities continuing to disproportionately affect the black African majority population as well as coloured populations[9]. In South Africa the term ‘coloured’ is used to identify a group of people with mixed black and white ethnicity who have a relatively distinct cultural identity, particularly in the Western Cape. In the apartheid era these racial categories were used by the government to legitimise state-sponsored oppression of ‘black’ and ‘coloured’ people. As the effect of these practices on health and access to resources may still be apparent, these categories are used within the context of the study to examine persisting inequalities in the post-apartheid era. Although the same categories are employed here, they are acknowledged as socially constructed and not as essentialist representations of ‘race’[12].

South Africa has also been described as one of the most ‘unequal societies in the world’[13]. Income inequality increased in the post-apartheid years, from 1993 to 2008[14], with rising inequality fuelled by an increased share of wealth going to the country’s wealthiest decile[14, 15]. It has been suggested that racial inequality remains interwoven with economic inequality[15], with the authors of a recent OECD report suggesting that *“levels of poverty and inequality continue to bear a persistent racial undertone”* [15].

Thus, the interplay of racial and material inequalities within South Africa in the post-apartheid era remains complex and may be associated with detrimental consequences for adolescent mental health. Although South Africa is a middle income country, on many major health indicators its outcomes are worse than those of lower income countries[9]. In countries experiencing greater income inequality, associations between poverty and poorer mental health may be stronger than in societies which are more equitable[1, 16]. The picture is further complicated by South Africa having a high prevalence of violence, with much of this accounted for through interpersonal and gender-based violence and children experiencing or witnessing high levels of violence and neglect[16]. These are important risk factors for adverse mental health[16, 17]. The high prevalence of violence may be related to underlying income inequality[16, 18].

With this in mind, we conducted an analysis of data from the South African Health and Well-being Study (SHaW). The study was broadly based on an earlier study of risk and protective factors for adolescent mental health carried out in a socially deprived multi-ethnic area of East London, UK, the Research with East London Adolescents: Community Health Survey (RELACHS)[19]. Its purpose was to address the knowledge gap in risk and protective factors for adolescent mental health in a different multi-ethnic setting from London, UK[19]. Our primary objective was to assess the association of poverty and disadvantage with psychological morbidity. In particular we hypothesised that children living in materially disadvantaged circumstances or from historically disadvantaged ethnic/racial backgrounds would be more likely to screen positive for common mental disorders (CMD) and Post Traumatic Stress Disorders (PTSD). As a second objective, we sought to identify important experiences such as racially-motivated bullying, levels of self-esteem, social support and exposure to violence, as potential mediators of observed associations, as previous work in this area has indicated their potential importance[1, 2, 17, 20, 21].

## Methods

### Survey design and participants

The study was conducted amongst eighth-graders (aged 14–15), studying in schools in Cape Town, South Africa, between March and September 2009. A pilot study was conducted to assess the reliability of the scales employed in the main survey[22]. Two schools were randomly chosen from one of three fee-paying strata (high, moderate, low). In order to increase the proportion of white children in the sample to reflect the demographic distribution in the Western Cape, a seventh school was added. This sampling procedure resulted in a school-fee stratified random sample, which was representative of all pupils attending schools in the large ‘Metro Central’ district of Cape Town. This part of Cape Town represents neighbourhoods which were previously demarcated as ‘white’ ‘coloured’ and ‘African’ residential areas.

Questionnaires were translated from English into the other two main languages spoken in Cape Town (Afrikaans and isiXhosa). Back translation of questionnaires in Afrikaans and isiXhosa was undertaken by native speakers. Questionnaires were completed by children in the language of their choice under exam conditions in class, ensuring confidentiality, within 60 minutes. The previous pilot study indicated that all scales employed within the survey had at least fair reliability[22].

Participants were asked to self-identify their race/ ethnicity according to the categories of black, white, coloured, Indian and ‘other’. These categories were similar to those employed in the South African census and also used in a previous nationally representative survey[23]. The use of these categories allows the possibility of assessing persistent health inequalities in people who have been historically disadvantaged[9, 12].

### Measures

#### Social disadvantage

Children were asked to report if their living quarters had one of the following: electricity, tap-water, television, indoor bathroom, a motorcar or bakkie (van), computer or internet access, or if their household received government grants. Children reporting two or more persons per room in their household (excluding kitchens, bathrooms and hallways) were classed as living in over-crowded accommodation[24]. These ‘durable assets and household amenities’, were used to derive an ‘asset index’ using a Principal Components Analysis (PCA),[25, 26] as described below. This approach to deriving an ‘asset index’ has been used in a number of LMIC contexts to derive a measure for wealth in the absence of income/ consumption data[25, 26]. The resulting asset index is internally coherent and has been validated against expenditure data across international settings[26] and closely matches data on consumption[27].

Children were asked if they could afford any of the following basic items: 3 meals per day, soap to wash daily, school uniform or equipment, clothes to keep warm and dry and more than one pair of shoes. Respondents were also asked if they could afford medicines and visits to their doctor. Responses to these questions were summed into a single ‘ability to afford basic items’ scale.

Children in the wrong grade for age (i.e. not aged 13–15) were classified as ‘educationally disadvantaged’. This was based on approaches taken by the investigators who derived the South African Index of Multiple Deprivation (SA IMD), which includes a domain for childhood educational deprivation[28]. Finally, parental employment status was enquired after.

### Mediators

The Multi-Dimensional Scale of Perceived Social Support (MSPSS)[29] assessed social support from family, friends and significant others. This instrument has been shown to have good concurrent, construct and discriminant validity and high internal and test re-test reliability[29, 30] and good reliability and validity with adolescents [31]. Responses to this questionnaire were summed and then categorised into quintiles. To assess for racially-motivated bullying, children reported how often they had been bullied because of their religion or race. Responses were dichotomised into ‘at least once this term’ versus ‘not this term’, (in the original question children were asked if they had been bullied that term (and the frequency of bullying over the term vs. no bullying at all)). Finally, self-esteem, was measured by 7 items from the Global Self Worth subscale of the Self Esteem Questionnaire (SEQ)[32], which has high validity and has adequate psychometric properties in South African adolescents[33]. Responses were summed and categorised into quintiles.

### Violence and PTSD

The Harvard Trauma Questionnaire[34] assessed for experiences of violence and Post-Traumatic Stress Disorder (PTSD), according to DSM-IIIR criteria. The questionnaire was adapted to reflect the context of South Africa and has previously been used successfully in this setting[17].

### Common mental disorders (CMD)

In community settings depression and anxiety disorders are typically grouped together and referred to as the *common mental disorders*[35]. This is valid as depressive and anxiety symptoms are highly comorbid and share similarities in core psychopathology[36–38]. Depression and anxiety also show similarities with respect to treatment and epidemiology[35, 36, 38].

The Short Moods and Feelings Questionnaire (SMFQ)[39], measuring core depressive symptoms, was used. This is a 13-item tool which has been internationally validated, with the pilot study indicating reasonable reliability[22]. Scores of 8 or more were taken as denoting that children were a case for depression[39]. The Zung self-rated anxiety scale was used to assess for anxiety[40]. This scale has previously been shown to have good internal reliability in South Africa[41]. Responses to 19 questions on this scale were summed, divided by the maximum score and multiplied by 100 to provide a total[22]. Children scoring 43 or more were classified as a ‘case’. Children meeting thresholds on either measure were classified as a ‘case’ for common mental disorders (CMD).

### Statistical methods

Analyses were conducted in Stata 13[42] and took into account the complex survey weightings, clustering and stratification of the dataset, using the *svy* suite of commands. An equal number of classes were selected in each school regardless of school size. Data were therefore reweighted to adjust for unequal probabilities of selection. Poisson regression using log-binomial models, with cluster robust estimation accounted for through the *pweight* function in Stata, was used to derive prevalence ratios or Relative Risk (RR) of association with 95% Confidence Intervals (95% CIs). Strength of association was assessed using design-based Wald tests in and, where relevant, used to assess for linear trends in associations, as well departures from linearity[43]. As there was no evidence in support of gender as an effect modifier in final models, all models adjusted for gender as a confounder.

Principal Components Analysis (PCA) was used to derive a single measure of wealth[25] or ‘asset index’ [25, 26, 44]. This approach uses a statistical approach to assign weights to household items which are unaffected by short-term fluctuations in family income[26]. The most unequally distributed assets are given the largest weights[25, 44]. Variables assessing ownership of durable assets and household amenities were used in PCA. Missing values were replaced with the mean based on approaches taken by previous investigators [25, 45, 46]. PCA using the correlation matrix, specifying one component, was used to derive a single measure. The derived variable was then grouped into quintiles[25].

To determine mediation, the following criteria had to be met (http://davidakenny.net/cm/mediate.htm#BK): 1. Main independent variables (ethnicity and material disadvantage) had to be correlated with dependent variables (CMD and PTSD); 2. Independent variables (ethnicity and material disadvantage) had to be correlated with putative mediators; 3. Putative mediators had to be associated with dependent variables (CMD and PTSD); 4. For complete mediation, the effect of independent variables (ethnicity and material disadvantage) on dependent variables (CMD and PTSD), had to reduce to a risk ratio of 1.00, after controlling for putative mediators (exposure to violence, racially-motivated bullying, self-esteem and social support) in regression models; If this last condition was not met whilst steps 1–3were met, then partial mediation was determined as having occurred.

### Ethical approval

Parents of children received written information about the study and were able to opt their children out of the study. Parents opted their children out of the study by returning a slip to the school. Parents were able to contact the school if they had questions. Provided parents of children had not opted them out, children present in school on the day of data collection were invited to participate and asked to provide written assent, using standardised forms which had been approved by an ethics committee. Ethical approval for the assent procedure and to conduct the study was granted by the University of Cape Town Research Ethics Committee and from the Western Cape Education Department, REC REF 037/2009.

## Results

1034 (of a total of 1169 possible students) took part, resulting in a response rate of 88%. Children not completing questionnaires were absent from school on the day of data collection. Levels of missing data for each variable were in general low with most variables having valid responses for 90% or more of participants. The derived variable for household overcrowding had the greatest amount of missing observations (data available for 80% of participants on this measure), probably because this variable was derived from two other measures (no. of people in household and no. of rooms in household).

Demographic features of the full sample are displayed in supporting information (S1 Table). Table 1 displays the distribution of disadvantage indicators across the sample, by race/ ethnicity. In general, children who described their race/ ethnicity as ‘black’ appeared more disadvantaged across most durable asset/household indicators. The strongest evidence of disadvantage was evident for measures indicative of ability to afford basic items. For example, black children reported that they were less likely to afford basic items such as soap to wash (15% versus 1.5% coloured children, 3.9% white children 0% other groups(*x*^*2*^ = 69.65;p = 0.04). More than a quarter of black children reported being unable to afford more than one pair of shoes (compared to 4.3% coloured children and 4.8% white children, (*x*^*2*^ = 112.63;p = 0.04)). 37.5% of black children were in a lower grade for their age, compared with 8.3% coloured children, 12% white children and 12% children reporting their ethnicity as ‘other’ (*x*^*2*^ = 117.97;p = 0.01). Maternal employment was broadly equivalent across the sample, although paternal unemployment was more likely for children reporting their ethnicity as ‘black’ (46.1%) or ‘other’ (38.5%) (vs. 14.3% of white children). The presence of household electricity was fairly universally distributed across the full sample, irrespective of ethnicity. Eight children describing themselves as belonging to an ‘other’ ethnic/ racial group appeared to be relatively disadvantaged across most of the measures.

**Table 1:** Disadvantage indicators by race/ ethnicity.

Race/ ethnicity	black	coloured	white	Indian	other	p value*
n (%)	268 (27)	612 (61)	101 (10)	21 (2)	8 (1)	
**Durable assets and household amenities**						
House type: shack/wendy vs brick/ other	31.5	7.2	5.9	0.0	0.0	0.10
no electricity	2.5	0.8	0.0	0.0	0.0	0.41
no tap water	8.4	1.8	2.9	0.0	0.0	0.20
no TV	5.8	1.0	3.9	0.0	0.0	0.23
no indoor bathroom	42.4	10.2	8.6	0.0	24.0	0.14
no motor car or bakkie (van)	52.7	26.1	11.7	0.0	38.6	0.20
no computer	71.5	28.6	14.0	5.4	38.7	0.05
no internet in the home	85.1	55.7	21.1	15.4	50.9	0.03
household receiving government grants	48.8	29.3	20.2	14.7	36.2	0.09
overcrowding (2+ persons/ room)	57.3	50.7	10.7	14.6	57.8	0.09
**Ability to afford basic amenities**						
can't afford 3 meals/ day	18.4	3.5	4.9	0.0	0.0	0.10
can't afford soap/ shampoo to wash everyday	15.0	1.5	3.9	0.0	0.0	0.04
can't afford school uniform	8.4	2.4	1.0	0.0	0.0	0.19
can't afford school equipment	16.6	2.1	2.9	0.0	26.7	0.07
can't afford clothes to keep warm and dry	30.7	3.4	6.7	0.0	12.0	0.03
can't afford visits to doctor and medicines	31.9	7.7	9.6	0.0	26.7	0.06
can't afford more than one pair of shoes	27.4	4.3	4.8	0.0	0.0	0.04
**Educational deprivation**						
In wrong grade for age	37.5	8.3	9.2	0.0	12.0	0.01
**Parental occupation**						
Father unemployed/ not living with father	46.1	26.6	14.3	4.7	38.5	0.09
Mother unemployed/ not living with mother	38.4	30.1	25.7	29.5	51.1	0.57

**Key:** Values in table are % unless stated otherwise

*from chi squared tests

PCA was used to derive a single asset index of durable assets and household amenities. The first component had an eigenvalue of 3.00 and accounted for 30% of variation in the original measures. Survey weighted means with 95% confidence intervals on this measure by race/ ethnicity were: white children:1.05 (-0.46,2.57); black children:-1.24 (-2.82,0.33); coloured children:0.40 (-0.49,1.28); Indian children:1.58 (1.10,2.06); ‘other’ children:-0.02 (-2.11,2.07) (lower values indicating greater disadvantage) (see S1 Fig which displays the distribution of the PCA-derived asset index by ethnicity and for the full sample).

In the full sample, the unadjusted prevalence of depression was 41% and anxiety was 16%, giving a combined prevalence for common mental disorders (anxiety or depression) of 47%. By self-ascribed ethnicity the prevalence of CMD was: 29% (white children), 58% (black children), 45% (coloured children), 24% (Indian children) and 41% (children self-classifying as ‘other’ ethnicity). The prevalence of PTSD in the full sample was 21% and by ethnicity: 14% (white children), 34% (black children) and 19% (coloured children).

Table 2 displays the unadjusted prevalence of CMD and PTSD according to race/ ethnicity and material disadvantage indicators. Broadly, across each of the racial/ ethnic groups, a gradient was evident in the association of increasing disadvantage, although both black and white children in the poorest fifth did not always follow this pattern. Put in another way, reduced access to resources in terms of ability to afford basic items or ownership of basic amenities and durable asset index was broadly associated with an increased prevalence of either CMD or PTSD. Broadly, for each level of disadvantage, the unadjusted prevalence of common mental disorders or PTSD was greater in black or coloured children than in white children (Table 2). There were too few observations to derive stable estimates for Indian children or children describing their racial/ ethnic group as ‘other’.

**Table 2:** Prevalence of CMD and PTSD.

Race/ ethnicity	white	black	coloured	Indian	other
n (%)	268 (27)	612 (61)	101 (10)	21 (2)	8 (1)
**CMD (%)**	28.7	57.7	44.5	24.0	41.0
**Afford basic items**					
Can afford all items	25.4	53.8	41.0	24.0	58.8
Can't afford one item	49.6	52.9	61.9	.	.
Can't afford 2+ items	43.2	68.5	69.1	.	.
**Asset index**					
first (richest) quintile	22.6	51.1	33.1	29.3	.
second quintile	28.1	49.7	38.0	24.2	.
third quintile	42.4	62.6	44.9	.	.
fourth quintile	71.5	68.7	55.5	.	44.8
fifth (poorest) quintile	25.3	51.3	56.0	.	.
**PTSD (%)**	13.6	33.8	19.0	0.0	0.0
**Afford basic items**					
Can afford all items	12.1	33.5	17.2	.	.
Can't afford one item	24.8	21.8	25.6	.	.
Can't afford 2+ items	19.9	41.7	35.5	.	.
**Asset index**					
first (richest) quintile	12.5	.	11.1	.	.
second quintile	22.5	33.3	21.4	.	.
third quintile	.	35.3	20.3	.	.
fourth quintile	.	40.6	18.3	.	.
fifth (poorest) quintile	16.4	30.9	23.4	.	.

**Key:** Numbers shown are percentages, unless stated otherwise. Prevalence estimates take into account survey weights.

There was strong evidence of an association between race/ ethnicity and exposure to violence or racially motivated bullying, with coloured children most exposed to violence, and Indian children most likely to be bullied because of religion or race. Exposure to the adverse mediators of racial bullying, poorer social support and lower self-esteem, each followed a linear trend with increasing material disadvantage/ reduced access to resources (S2 Table).

Table 3 displays the relative risk for association of independent variables with CMD and PTSD, after adjusting for gender. After adjusting for gender, both CMD and PTSD were more than twice as likely in black children, relative to white children and CMD was 1.73 times more prevalent in coloured children (95% CI:1.11, 2.70). Estimates for PTSD were not available for Indian and ‘other’ children, due to small numbers in cells. There was a dose-response relationship for the summed ‘ability to afford basic items’ measure with both CMD and PTSD. As access to healthcare may be distinct from access to financial resources we repeated analyses using the scale which assessed ‘ability to afford basic items’ with healthcare measures taken out. Associations persisted and were similar, after the question on ‘ability to afford medicines and visits to the doctor’ was removed from the ‘ability to afford basic items’ scale *(Per one item increase for CMD this was RR*: *1*.*17 (95% CI*: *1*.*07*, *1*.*28) p = 0*.*009 (trend)*, *for PTSD this was RR*: *1*.*23(95% CI*:*1*.*06*, .*42) p = 0*.*02 (trend))*. After adjusting for gender, children who reported not being able to afford medicines or visits to their doctor were 1.17 (95% CI: 1.06, 1.29) times more likely to screen positive for CMD and 1.23 times more likely (95% CI: 1.05, 1.45) to screen positive for PTSD. Most of the putative mediators were associated with CMD and PTSD (Table 3). Associations between most of the putative mediators and CMD and PTSD persisted after adjustment of ethnicity and all socioeconomic position/ disadvantage indicators (S3 Table).

**Table 3:** Gender-adjusted association of individual disadvantage indicators with CMD and PTSD. Relative Risk (RR) and 95% Confidence Intervals (95% CIs).

		CMD		PTSD
	RR	(95% CI)	RR	(95% CI)
**Race/ ethnicity**				
White	1.00	REF	1.00	REF
Black	2.27	(1.24, 4.15)	2.21	(1.73, 2.83)
Coloured	1.73	(1.11, 2.70)	1.32	(0.92, 1.90)
Indian	0.96	(0.66, 1.39)	.	.
Other	1.72	(0.57, 5.17)	.	.
**Asset index (quintiles)**				
Wealthiest quintile	1.00	REF	1.00	REF
second quintile	1.32	(0.75, 2.35)	2.19	(2.04, 2.35)*
third quintile	1.52	(0.80, 2.90)	2.03	(1.43, 2.88)
fourth quintile	1.98	(0.98, 4.00)	2.45	(1.44, 4.16)
Poorest quintile	1.72	(0.91, 3.24)	2.72	(1.92, 3.85)
**Educational deprivation**				
Wrong grade for age	1.42	(0.90, 2.23)	1.44	(0.61, 3.38)
**Cannot afford basic items**				
Per one item increase (*continuous)*	1.14	(1.05, 1.25)*	1.20	(1.05, 1.38)*
**Parental employment**				
Father unemployed/ not living with father	1.33	(0.87, 2.03)	1.42	(0.92, 2.21)
Mother unemployed/ not living with mother	1.13	(0.79, 1.64)	1.22	(0.86, 1.71)
**Mediators**				
Per one unit reduction in social support (quintiles)	1.27	(1.13, 1.42)**	1.17	(0.94, 1.46)
Per one unit reduction in self-esteem (quintiles)	1.32	(1.18, 1.48)**	1.32	(1.25, 1.40)***
Discrimination- Made fun of because of religion or race at least once this semester (vs. not at all)	1.52	(1.21, 1.91)	1.47	(1.00, 2.16)
Exposure to 1+ violent incident (vs. none)	2.19	(0.94, 5.11)	3.75	(0.85, 16.62)

**Key:** Tests for linear trend

*p<0.05;

**p<0.01;

***p<0.001.

All models are gender-adjusted.

After adjusting for disadvantage indicators and ethnicity in models, most associations of disadvantage with common mental disorders were attenuated. After adding putative mediators to models, most associations of ethnicity and disadvantage with CMD were partially attenuated, supporting at least partial mediation by these variables (Table 4). Of note, however, black and coloured children continued to experience an elevated relative risk of CMD, compared to white children, even after adjusting for potential mediators (Table 4) and children who reported experiencing violence were more than twice as likely to screen positive for CMD in adjusted models (Table 4) (RR:2.39 (95% CI:1.22,4.68; p<0.001). As with the analyses concerning common mental disorders, most associations for PTSD were attenuated with the addition of potential mediators. Overall, associations with race/ ethnicity and disadvantage indicators were less marked with PTSD than with CMD, in adjusted models (Table 4).

**Table 4:** Multivariable associations with Common Mental Disorders (CMD) (n = 583) & Post Traumatic Stress Disorders (PTSD) (n = 532); Relative Risk (RR) and 95% Confidence Intervals (95% CIs).

		CMD				PTSD		
		Model 1		Model 2		Model 1		Model 2
	RR	(95% CI)	RR	(95% CI)	RR	(95% CI)	RR	(95% CI)
**DISADVANTAGE INDICATORS**								
**Race/ ethnicity**								
White	1.00	REF	1.00	REF	1.00	REF	1.00	REF
Black	1.68	(1.08, 2.60)	1.48	(1.04, 2.10)	1.44	(0.72, 2.89)	1.32	(0.68, 2.56)
Coloured	1.64	(1.44, 1.86)	1.44	(1.23, 1.69)	1.02	(0.54, 1.91)	0.92	(0.49, 1.75)
Indian	1.24	(0.93, 1.64)	1.38	(0.87, 2.20)	.	.	.	.
‘other’	1.41	(0.55, 3.61)	1.56	(0.39, 6.16)	.	.	.	.
**Asset index**								
richest quintile	1.00	REF	1.00	REF	1.00	REF	1.00	REF
second quintile	1.03	(0.54, 1.98)	0.92	(0.62, 1.38)	1.82	(1.30, 2.54)	1.63	(1.33, 2.01)
third quintile	1.17	(0.70, 1.95)	0.99	(0.67, 1.46)	1.78	(1.01, 3.13)	1.57	(0.99, 2.47)
fourth quintile	1.38	(0.68, 2.80)	1.02	(0.59, 1.77)	1.54	(0.44, 5.33)	1.16	(0.37, 3.67)
poorest quintile	1.12	(0.55, 2.30)	0.87	(0.53, 1.42)	1.62	(0.51, 5.19)	1.31	(0.50, 3.45)
**Educational deprivation**								
wrong grade for year	1.13	(0.65, 1.96)	1.12	(0.80, 1.56)	0.96	(0.33, 2.81)	0.89	(0.37, 2.13)
**Cannot afford basic items (continuous)**	1.10	(0.97, 1.24)	1.03	(0.93, 1.15)	1.15	(0.95, 1.40)	1.10	(0.96, 1.25)
**Parental employment**								
father unemployed	1.13	(0.85, 1.49)	1.09	(0.76, 1.56)	1.13	(0.56,2.28)	1.11	(0.54, 2.29)
mother unemployed	0.94	(0.78, 1.13)	1.02	(0.83, 1.24)	0.94	(0.54,1.63)	1.02	(0.65, 1.60)
**MEDIATORS**								
decreasing social support *(continuous- per quintile)*	-	-	1.09	(1.00, 1.19)	-	-	1.02	(0.92, 1.15)
decreasing self-esteem *(continuous-per quintile)*	-	-	1.29	(1.14, 1.45)		-	1.28	(1.06, 1.54)
made fun of because of religion or race	-	-	1.20	(0.89, 1.62)	-	-	1.27	(0.78, 2.06)
exposure to 1+ violent incident	-	-	2.39	(1.22, 4.68)		-	2.51	(0.43, 14.52)

**Key:** Model 1: Adjusted for gender, disadvantage indicators, Model 2: Adjusted for gender, disadvantage indicators and mediators

## Discussion

### Main findings

Despite high level dismantling of the apartheid system, this study demonstrates that fundamental structural social inequalities remained, 15 years after the demise of apartheid. Although some things may have improved for the health and wellbeing of the least advantaged groups in South Africa, the evidence for this was not shown by our study. The legacy of apartheid inequalities are still potentially observable in the inequitable mental health status of young South Africans.

There was a high unadjusted prevalence of common mental disorders in this representative sample of adolescents growing up in Cape Town, South Africa. The prevalence of CMD and PTSD are higher than previously reported estimates of between 15.2–16.0% in urban samples from townships in the Western Cape of South Africa (Khayelitsha) [47], and also higher than those reported in the South African adult mental health surveys[48]. This may have been due to sub-threshold symptoms being included in estimates[49], or reflect a true higher prevalence of mental disorder in this population. In general, there was evidence of a gradient in the association of material disadvantage with CMD/ PTSD across all racial/ ethnic groups, although a slight departure from this was noted for the poorest fifth on the asset index, for white and black children. This may have been due to a number of factors, including out-migration from the most extreme levels of poverty, or artefact due to reduced sample size affecting the poorest fifth. These findings are consistent with the wider literature on poverty and common mental disorders, in LMIC settings[1], indicating that material disadvantage has a strong association with increased CMD/PTSD. The levels of poverty, discrimination, violence and childhood adversity reported within the present study is similar to that reported previously [9, 17, 21, 50–53].

Racial/ ethnic disadvantage with respect to most material disadvantage indicators, some of the potential mediators (experiences of violence, racial bullying) and prevalence of mental ill-health, remains elevated in children who would have been discriminated against in South Africa under apartheid[9, 52]. It is noteworthy that such stratifications persist in children mostly born in the year that apartheid ended. Access to amenities such as electricity were nearer universal in the sample, indicative of some improvements to living standards recently; which have also been noted elsewhere[9, 52]. However, the findings suggest that the legacy of inequality persists for other measures. In particular, black children appeared the most disadvantaged across almost all poverty indicators. Whilst the gradient for the association of poverty indicators with adverse mental health outcomes was observed in all children, there was a higher prevalence of CMD and PTSD for black and coloured children for each level of material disadvantage. In addition, although the association of material disadvantage indicators (ability to afford basic items, asset index, educational deprivation and parental employment) with common mental disorders and PTSD had diminished after adjustment for putative mediators in final models, (suggestive of full mediation by these variables), black and coloured children continued to have a higher risk of common mental disorders after adjustment for all putative mediators and material disadvantage indicators (with similar trends for Indian children and children reporting their race/ethnicity as 'other'), relative to white children. This may suggest that there are additional factors which we were not able to account for in our models, which may play a role in patterning the excess risk of common mental disorders in children of these ethnic/racial groups. More research is clearly needed, however these findings support prior observations from other reports that material disadvantage and poverty remain intertwined with racial/ethnic inequalities, within this context [9, 15].

These findings are consistent with other reports[9, 50, 52] indicating great challenges in tackling the structural inequalities underlying the social determinants of health within this context[54], and specifically the distribution of resources and life chances which may continue to follow deeply entrenched social stratifications[9, 12].

## Strengths and limitations

Strengths of the survey include its high response rates with low levels of missing data reducing non-response bias. In addition, the sampling frame led to an ethnically and socio-economically diverse sample, representative of pupils attending schools within a large metropolitan area of Cape Town. This permitted the assessment of disadvantage across multiple domains, as well as an estimation of prevalence of CMD and PTSD. Most measures have adequate validity and reliability in this or other equivalent settings, confirmed by a prior pilot study conducted within this setting[22]. Use of materials in languages other than English has meant that assessment would have been less prone to measurement bias.

Limitations of the survey include its cross-sectional design, limiting inferences around direction of association and the possibility of reverse causality in some of the measures, for example children who are currently depressed may also have low self-esteem as a result of a depressed mental state. In addition, although the sample was representative of a defined region in Cape Town, the generalisability of findings to other parts of South Africa remains unclear, further work is needed to establish how the findings reported here differ from other parts of South Africa. It is clear that mental disorders in adolescence have multifactorial origins, associated with, but not confined to, poverty and social disadvantage [2]. It was not possible to adjust for these other indicators,(which may include stressful life events and academic stress, family history of mental illness and living with a parent with a mental disorder[2]). It is possible that these, as well as other variables, played a part in the findings which we could not account for. Our analyses also treated gender as a confounder as there was no statistical evidence in our analyses in support of effect modification by gender. Previous work has suggested that types of mental disorder experienced in adolescence also vary by gender[2]. The multifactorial nature of adolescent mental disorders within this context, including the role of gender in accounting for associations, should be investigated in future work.

## Future research and policy

Future research should focus on the inter-play of poverty with racial/ ethnic disparities[9], and longitudinal analyses of the associations between material disadvantage and mental health among adolescents in low and middle-income countries such as South Africa. Our study focused on the experience of adolescent learners within multi-ethnic schools in South Africa. Future work could also take into account the role of the school context (for example ethnic composition) in accounting for mental health in schools where children form a relative minority[55]. Larger nationally representative cohort studies that follow children through adolescence into adulthood are likely to yield more specific information on the complex mechanisms underlying the associations between material advantage, mediating variables (such as violence, social support, bullying and self-esteem) and mental health, observed in this study. Further work on the development of valid and reliable mental health and socio-economic measures is integral to this task[56]. In addition, there is an urgent need for studies that evaluate the impact of interventions on two hypothesised mechanisms of the poverty-mental health association in LMIC: firstly population-level intervention studies which address the root causes of material and social inequalities[1, 54] and assess their impact on mental health disparities; and secondly mental health intervention studies that evaluate the effect of a wide range of prevention and treatment interventions on material and educational disadvantage over time[57]. From a policy perspective, this study presents compelling evidence that mental health is integrally linked with material and ethnic disadvantage among adolescents in South Africa, and should be included in poverty alleviation interventions targeting young people.

## In conclusion

In conclusion, this report confirms disparities in the mental health of young people growing up in South Africa, 15 years after the demise of apartheid. These findings raise some concern given the known continuity of childhood mental health with adult mental health[6] as well as the role which childhood disadvantage and childhood psychological morbidity plays in a range of adverse downstream health and social outcomes[58–60].

## Supporting Information

S1 Fig Distribution of single asset indicator by ethnicity. (DOCX)

S1 Table Demographic features of the sample. (DOCX)

S2 Table Distribution of mediators by ethnicity and material disadvantage. (DOCX)

S3 Table Association of mediators with CMD and PTSD after adjustment for disadvantage indicators and gender. (DOCX)
