# Supplementary material for: Mental Health Inequalities in Adolescents Growing Up in Post-Apartheid South Africa: Cross-Sectional Survey, SHaW Study
Source: PLoS One. 2016 May 3;11(5):e0154478. doi: 10.1371/journal.pone.0154478 (PMC4854374; doi:10.1371/journal.pone.0154478)
Supplement: S1 Fig — (DOCX) [file pone.0154478.s001.docx]

**S1 Figure: Distribution of single asset indicator by ethnicity**
