# Supplementary material for: Mental Health Inequalities in Adolescents Growing Up in Post-Apartheid South Africa: Cross-Sectional Survey, SHaW Study
Source: PLoS One. 2016 May 3;11(5):e0154478. doi: 10.1371/journal.pone.0154478 (PMC4854374; doi:10.1371/journal.pone.0154478)
Supplement: S1 Table — (DOCX) [file pone.0154478.s002.docx]

**S1 Table: Demographic features of the sample**

|  |  | **n** | **%** |
| --- | --- | --- | --- |
| **Race/ ethnicity** | Black | 268 | 26.5 |
|  | Coloured | 612 | 60.6 |
|  | White | 101 | 10 |
|  | Indian | 21 | 2.1 |
|  | Other | 8 | 0.8 |
| **Gender** | Male | 433 | 46.1 |
|  | Female | 507 | 53.9 |
| **Asset index (quintiles)** | (Richest) quintile | 178 | 17.2 |
|  | Second quintile | 232 | 22.5 |
|  | Third quintile | 198 | 19.2 |
|  | Fourth quintile | 219 | 21.2 |
|  | (Poorest) quintile | 207 | 20 |
| **Educational deprivation** | In wrong grade for age | 154 | 16 |
| **Durable assets and household amenities** | House type: shack/wendy vs brick/ other | 139 | 13.7 |
|  | No electricity | 14 | 1.3 |
|  | No tap water | 37 | 2.7 |
|  | No TV | 27 | 2.7 |
|  | No indoor bathroom | 188 | 18.4 |
|  | No motor car/ bakkie | 318 | 31.3 |
|  | No computer | 387 | 37.4 |
| **Ability to afford basic items** | Can't afford 3 meals/ day | 78 | 7.6 |
|  | Can't afford soap/ shampoo to wash | 57 | 5.6 |
|  | Can't afford school uniform | 42 | 4.1 |
|  | Can't afford school equipment | 66 | 6.4 |
|  | Can't afford clothes to keep warm and dry | 113 | 11.1 |
|  | Can't afford visits to doctor and medicines | 151 | 14.9 |
|  | Can't afford more than one pair of shoes | 108 | 10.6 |
| **Parental occupation** | Father unemployed/ not living with father | 286 | 29.9 |
|  | Mother unemployed/ not living with mother | 307 | 32.2 |

*All estimates take survey weights into account*
