# Supplementary material for: Mental Health Inequalities in Adolescents Growing Up in Post-Apartheid South Africa: Cross-Sectional Survey, SHaW Study
Source: PLoS One. 2016 May 3;11(5):e0154478. doi: 10.1371/journal.pone.0154478 (PMC4854374; doi:10.1371/journal.pone.0154478)
Supplement: S2 Table — (DOCX) [file pone.0154478.s003.docx]

**S2 Table**

**Distribution of mediators by race/ ethnicity and material disadvantage**

|  |  |  | |  |  | **Race/ ethnicity** |  |  |  |  |  | **Asset index** |
| --- | --- | --- | --- | --- | --- | --- | --- | --- | --- | --- | --- | --- |
|  | **black** | **coloured** | | **white** | **Indian** | **other** |  | **richest quintile** | **second quintile** | **third quintile** | **fourth quintile** | **poorest quintile** |
| **n (%)** | **268 (27)** | **612 (61)** | | **101 (10)** | **21 (2)** | **8 (1)** |  | **178 (17)** | **232 (22)** | **198 (19)** | **219 (21)** | **207 (21)** |
| **Exposed to violence** (%) | 82 | 88 | | 68 | 47 | 87 |  | 69 | 83 | 90 | 91 | 86 |
|  |  |  | |  |  | *p=0.004^*^* |  |  |  |  |  | *p=0.26^‡^* |
| **Bullied because of religion/**  **race** (%) | 31 | 14 | | 22 | 37 | 30 |  | 14 | 19 | 14 | 29 | 25 |
|  |  |  | |  |  | *p=0.02^*^* |  |  |  |  |  | *p=0.005^‡^* |
| **Social support** |  |  | |  |  |  |  |  |  |  |  |  |
| (mean) | 58 | 64 | | 68 | 72 | 67 |  | 70 | 68 | 65 | 58 | 56 |
| [95% CI] | [53,62] | [61,68] | | [59,77] | [68,77] | [47,86] |  | [63, 76] | [64, 72] | [61, 68] | [54, 62] | [55, 58] |
|  |  |  | |  |  | *p=0.11^†^* |  |  |  |  |  | *p<0.001^‡^* |
| **Self esteem** | | | | | | |  |  |  |  |  |  |
| (mean) | 23 | | 24 | 25 | 25 | 24 |  | 25 | 24 | 24 | 23 | 23 |
| [95% CI] | [22,24] | | [24,25] | [23,27] | [23,27] | [20,29] |  | [25, 26] | [24, 25] | [23,25] | [22, 24] | [21, 24] |
|  |  | |  |  |  | *p=0.68^†^* |  |  |  |  |  | *p=0.001^‡^* |

***Key:*** *^*^ chi squared tests; ^†^one way analysis of variance; ^‡^test for linear trend*
