# Supplementary material for: Mental Health Inequalities in Adolescents Growing Up in Post-Apartheid South Africa: Cross-Sectional Survey, SHaW Study
Source: PLoS One. 2016 May 3;11(5):e0154478. doi: 10.1371/journal.pone.0154478 (PMC4854374; doi:10.1371/journal.pone.0154478)
Supplement: S3 Table — (DOCX) [file pone.0154478.s004.docx]

**S3 Table: Association of mediators with CMD and PTSD after adjustment for disadvantage indicators and gender**

**1. Association of mediators with common mental disorders**

|  |  | **RR** | **95% CI** |  | **RR** | **95% CI** |  | **RR** | **95% CI** |  | **RR** | **95% CI** |
| --- | --- | --- | --- | --- | --- | --- | --- | --- | --- | --- | --- | --- |
|  |  |  |  |  |  |  |  |  |  |  |  |  |
| **Mediator** | Social support (per quintile) | 1.23 | 1.13,1.33 | Self-esteem (per quintile) | 1.30 | 1.14,1.47 | Bullied because of race/ religion | 1.36 | 1.09,1.70 | Exposure to violence | 2.08 | 0.90,4.79 |
|  |  |  |  |  |  |  |  |  |  |  |  |  |
| Gender (female) |  | 1.51 | 1.08,2.10 |  | 1.28 | 0.99,1.66 |  | 1.35 | 1.01,1.81 |  | 1.38 | 1.07,1.79 |
| Asset index (per quintile) |  | 1.04 | 0.94,1.15 |  | 1.03 | 0.89,1.20 |  | 1.05 | 0.94,1.18 |  | 1.02 | 0.94,1.11 |
| Educational deprivation |  | 1.20 | 0.82,1.75 |  | 1.13 | 0.84,1.52 |  | 1.20 | 0.84,1.71 |  | 1.18 | 0.85,1.65 |
| Cannot afford basic items (per item) |  | 1.04 | 0.93,1.17 |  | 1.03 | 0.94,1.13 |  | 1.06 | 0.95,1.20 |  | 1.07 | 0.96,1.20 |
| Father unemployed |  | 1.09 | 0.92,1.30 |  | 1.11 | 0.89,1.39 |  | 1.19 | 0.89,1.60 |  | 1.15 | 0.95,1.38 |
| Mother unemployed |  | 0.96 | 0.73,1.27 |  | 0.98 | 0.80,1.21 |  | 0.95 | 0.81,1.12 |  | 1.00 | 0.77,1.31 |
| Race/ ethnicity |  |  |  |  |  |  |  |  |  |  |  |  |
| white |  | 1.00 | REF |  | 1.00 | REF |  | 1.00 | REF |  | 1.00 | REF |
| black |  | 1.56 | 1.14,2.12 |  | 1.72 | 1.02,2.89 |  | 1.64 | 1.28,2.11 |  | 1.67 | 1.12,2.49 |
| coloured |  | 1.57 | 1.27,1.94 |  | 1.62 | 1.21,2.18 |  | 1.69 | 1.30,2.19 |  | 1.52 | 1.09,2.13 |
| Indian |  | 1.18 | 0.91,1.52 |  | 1.09 | 0.89,1.34 |  | 1.12 | 0.90,1.40 |  | 1.22 | 0.94,1.57 |
| other |  | 2.13 | 0.95,4.79 |  | 1.63 | 0.41,6.54 |  | 1.22 | 0.46,3.23 |  | 1.51 | 0.44,5.24 |

**2. Association of mediators with Post Traumatic Stress Disorder**

|  |  | **RR** | **95% CI** |  | **RR** | **95% CI** |  | **RR** | **95% CI** |  | **RR** | **95% CI** |
| --- | --- | --- | --- | --- | --- | --- | --- | --- | --- | --- | --- | --- |
|  |  |  |  |  |  |  |  |  |  |  |  |  |
| **Mediator** | Social support (quintiles) | 1.15 | 0.99,1.35 | Self-esteem (quintiles) | 1.29 | 1.18,1.40 | Bullied because of race/ religion | 1.33 | 0.95,1.86 | Exposure to violence | 3.26 | 0.60,17.79 |
|  |  |  |  |  |  |  |  |  |  |  |  |  |
| Gender (female) |  | 1.23 | 0.76,1.98 |  | 1.06 | 0.77,1.47 |  | 1.12 | 0.72,1.75 |  | 1.19 | 0.82,1.73 |
| Asset index (per quintile) |  | 1.01 | 0.81,1.27 |  | 1.04 | 0.84,1.28 |  | 1.06 | 0.79,1.44 |  | 0.99 | 0.82,1.21 |
| Educational deprivation |  | 1.09 | 0.39,3.04 |  | 1.03 | 0.52,2.05 |  | 1.01 | 0.47,2.16 |  | 1.05 | 0.49,2.26 |
| Cannot afford basic items (per item) |  | 1.10 | 0.87,1.39 |  | 1.05 | 0.86,1.27 |  | 1.10 | 0.86,1.41 |  | 1.10 | 0.92,1.32 |
| Father unemployed |  | 1.23 | 0.66,2.27 |  | 1.13 | 0.76,1.68 |  | 1.11 | 0.73,1.68 |  | 1.21 | 0.85,1.72 |
| Mother unemployed |  | 0.92 | 0.53,1.60 |  | 1.01 | 0.64,1.60 |  | 0.98 | 0.61,1.56 |  | 1.02 | 0.62,1.65 |
| Race/ ethnicity |  |  |  |  |  |  |  |  |  |  |  |  |
| white |  | 1.00 | REF |  | 1.00 | REF |  | 1.00 | REF |  | 1.00 | REF |
| black |  | 1.49 | 0.84,2.62 |  | 1.71 | 1.16,2.53 |  | 1.59 | 0.91,2.78 |  | 1.51 | 0.88,2.60 |
| coloured |  | 1.12 | 0.63,1.99 |  | 1.31 | 0.77,2.23 |  | 1.30 | 0.65,2.56 |  | 1.18 | 0.69,2.02 |
| Indian |  | - | - |  | - | - |  | - | - |  | - | - |
| other |  | - | - |  | - | - |  | - | - |  | - | - |

*- No estimates as too few observations*
